# Separation and Extraction of Compound-Fault Signal Based on Multi-Constraint Non-Negative Matrix Factorization

**DOI:** 10.3390/e26070583

**Published:** 2024-07-09

**Authors:** Mengyang Wang, Wenbao Zhang, Mingzhen Shao, Guang Wang

**Affiliations:** 1 Changchun Institute of Optics, Fine Mechanics and Physics, Chinese Academy of Sciences, Changchun 130033, China; zhangwenbao@ciomp.ac.cn (W.Z.); mingzhenshao@163.com (M.S.); wangguang@ciomp.ac.cn (G.W.); 2 University of Chinese Academy of Sciences, Beijing 100049, China

**Keywords:** multi-constraint non-negative matrix factorization, underdetermined blind source separation, time–frequency distribution, parameter WK, compound fault diagnosis

## Abstract

To solve the separation of multi-source signals and detect their features from a single channel, a signal separation method using multi-constraint non-negative matrix factorization (NMF) is proposed. In view of the existing NMF algorithm not performing well in the underdetermined blind source separation, the β-divergence constraints and determinant constraints are introduced in the NMF algorithm, which can enhance local feature information and reduce redundant components by constraining the objective function. In addition, the Sine-bell window function is selected as the processing method for short-time Fourier transform (STFT), and it can preserve the overall feature distribution of the original signal. The original vibration signal is first transformed into time–frequency domain with the STFT, which describes the local characteristic of the signal from the time–frequency distribution. Then, the multi-constraint NMF is applied to reduce the dimensionality of the data and separate feature components in the low dimensional space. Meanwhile, the parameter WK is constructed to filter the reconstructed signal that recombined with the feature component in the time domain. Ultimately, the separated signals will be subjected to envelope spectrum analysis to detect fault features. The simulated and experimental results indicate the effectiveness of the proposed approach, which can realize the separation of multi-source signals and their fault diagnosis of bearings. In addition, it is also confirmed that the proposed method, juxtaposed with the NMF algorithm of the traditional objective function, is more applicable for compound fault diagnosis of the rotating machinery.

## 1. Introduction

The signal analysis of vibration in rotating machinery has been widely used in the field of fault diagnosis because the signals contain the operational state of the equipment [1,2]. However, in the case of the limitations on the number and installation location of sensors, the information obtained from the signals is limited [3,4]. Moreover, the non-stationary nature of the collected signals, the interference between multi-source fault signals and environmental noise may often result in the disappearance of feature information. Therefore, it is of great significance for the separation and extraction of compound faults based on vibration analysis [5,6].

There are many analysis methods based on vibration signals, such as feature extraction, pattern recognition and deep learning. For example, Wang et al. [7] proposed a fault diagnosis method based on sparsity-guided empirical wavelet transform, which can defect single and multiple fault bearings of railway axles. Lu et al. [8] introduced a method combining wavelet transform and K-mean clustering to realize the prediction about the battery state of health. Alimardani et al. [9] present an approach based on vibration signals to diagnose the faults of rotor eccentricity. Zhang et al. [10] developed a method based on the local outlier factor and improved adaptive matching pursuit, which can detect and recover the anomalous vibration signal. Li et al. [11] present an adaptive data fusion strategy based on deep learning with the convolutional neural network, which is validated on an industrial fan system with non-manufacturing faults and a centrifugal pump. Łuczak [12] proposed a method named CWTx6-CNN, which offered a clear representation of fault-related features. Wang et al. [13] introduced a novel fault recognition method on the basis of multi-sensor data fusion and bottleneck layer optimized convolutional neural network (MB-CNN) and realized the identification and classification of multiple faults of bearings. We know that analysis methods based on vibration signals mostly focus on low-dimensional analysis [14], and the information obtained from the original signal is bounded. It requires us to perform dimensionality transformation on one-dimensional vibration signals and observe the multi-dimensional signal so as to reveal unclear information. Simultaneously, the local feature information can be enhanced significantly with dimensionality transformation [15,16].

In the past few decades, many methods of dimensionality transformation have been proposed and widely applied in the fields such as signal separation, image clustering, biological information extraction, behavior feature recognition, and environmental perception and prediction [17,18,19,20]. The methods regarding dimensionality transformation can not only reduce the dimensionality of data but also extract salient features from high-dimensional data effectively. Meanwhile, it is beneficial for subsequent data processing and can achieve low dimensional visualization of data. The traditional dimensionality transformation algorithm actually seeks the intrinsic linear structure of the data in low dimensional space [21,22]. However, most of the internal structures of data are complex and show nonlinear characteristics. In addition, the dimensions of various types of data continue to grow at an extremely fast pace. Therefore, exploring the effective features and improving the ability to analyze such data has a positive effect. Machine learning algorithms based on matrix factorization are the key technologies for several types of problems in this field, including dictionary learning, non-negative matrix factorization (NMF), concept factorization, matrix padding, etc. [23,24,25]. Among them, the NMF algorithm has attracted much attention in feature extraction engineering due to its unique advantage of interpretability and scalability [26]. For example, Zhang et al. [27] proposed a weighted NMF algorithm, which achieved image clustering by optimizing three parameters in the algorithm. Gu et al. [28] introduced a method combining an improved NMF algorithm and a global position system to identify the sources driving ground deformation. Luo et al. [29] developed a novel approach based on the robust ensemble manifold projective NMF algorithm for image representation. Saha et al. [30] used a privacy-preserving NMF algorithm to ensure the degree of privacy guarantees. Li et al. [31] adopted a deep autoencoder-like NMF method for link prediction. In addition, the NMF algorithm performs well in the field of biomedicine. Marta et al. [32] proposed a negative binomial NMF algorithm, which can capture the variation across patients to extract the mutational signatures. Tu et al. [33] proposed a hypergraph regularized joint deep semi-NMF algorithm to identify biomarkers of Alzheimer’s disease. Nasrin et al. [34] put forward a model on the basis of the improved NMF algorithm that can recognize native decoys in protein structure prediction.

It can be observed that the NMF algorithm has been applied in many fields and has achieved many remarkable results since it was proposed. However, there is still some room to improve the NMF algorithm, especially in the blind source separation problem related to the diagnosis of compound faults in rotating machinery. Therefore, to solve the separation of multi-source signals and detect their features from a single channel, a signal separation method based on multi-constraint NMF algorithm is proposed. By utilizing the flexibility of *β*-divergence and the uniqueness of determinant constraint on the feature matrix, the objective function of non-negative matrix factorization can be converted to the minimum value smoothly, quickly and stably. According to the advantage of dimensionality transformation with the STFT algorithm, multi-constraint NMF algorithm, and construction of parameter WK, the proposed method can accomplish the separation of multi-source signals and their fault diagnosis of bearings, which makes fault diagnosis much easier and more reliable. As rolling bearings are important components of rotating machinery, this paper takes rolling bearings as the research object.

The remaining sections are organized as follows: Section 2 describes the basic principle of the NMF algorithm. The STFT algorithm, multi-constraint NMF algorithm and the parameter WK are introduced in Section 3. In Section 4, the specific separation of compound fault signals based on the suggested method is presented. The simulated and experimental results are discussed in Section 5. Finally, the conclusions are summarized in Section 6.

## 2. Principle of Non-Negative Matrix Factorization

**Figure 1:**
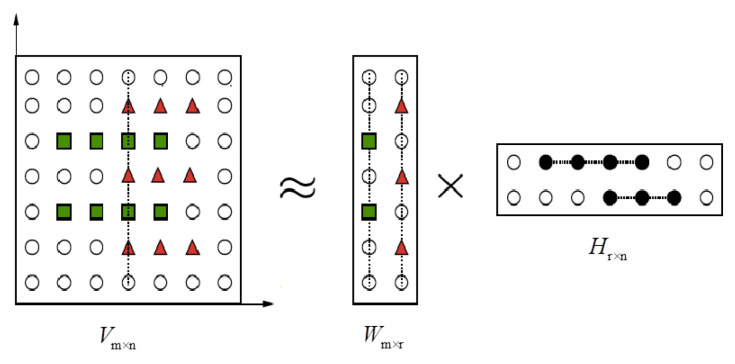
The model of the NMF algorithm.

The basic idea of the non-negative matrix factorization algorithm can be generally represented as follows: for any non-negative matrix $V\in R_{+}^{m\times n}$, the NMF algorithm is constructed with an approximate factorization of two non-negative matrices $W\in R_{+}^{m\times r}$ and $H\in R_{+}^{r\times n}$ [35], namely:

(1) $$V_{m\times n}\approx W_{m\times r}H_{r\times n}$$

where $V_{m\times n}$ denotes a matrix with the dimension of *m*, whereas n represents the number of samples. $W_{m\times r}$ denotes a basis matrix that can be regarded as a series of basis vectors. $H_{r\times n}$ denotes a coefficients matrix that can be regarded as the coordinates of each sample with respect to these basis vectors. In order to achieve better results of dimensionality reduction, the parameter r (rank of the matrix) is regarded as r<mn/(m+n). The model of the NMF algorithm is shown in Figure 1. In the field of signal processing, it can be explained that if each column of the matrix $V_{m\times n}$ is considered an observed signal, each group of observed signals contains different features (mixed features, single features, or redundant information) represented by green squares and red triangles. Each column of the matrix $W_{m\times r}$ contains the separated feature of the observed signal by the NMF algorithm, which can be reconstructed to the original signal by multiplying the coefficients matrix $H_{r\times n}$. It shows the idea of representing the whole based on parts.

At present, a variety of optimization algorithms about cost function are widely used, and the Euclid Distance is one of the most popular methods, which can be represented:

(2) $$\begin{array}{l} D(V||WH)={\left\|V-WH\right\|}^{2}\\ s.t.\;W,H>0 \end{array}$$

The cost function of Equation (2) is regarded as the following optimization problem:

(3) $$\min\;{\left\|V-WH\right\|}_{F}^{2}=\sum_{ij}[v_{ij}-{\left(WH\right)}_{ij}{]}^{2}$$

The above problem can be solved with a gradient descent algorithm until convergence. The updated rules are presented:

(4) $$w_{ik}\leftarrow w_{ik}\frac{{\left(VH^{T}\right)}_{ik}}{{\left(WHH^{T}\right)}_{ik}}\;,h_{kj}\leftarrow h_{kj}\sum_{i}\frac{{\left(W^{T}V\right)}_{kj}}{{\left(W^{T}WH\right)}_{kj}}$$

## 3. Basic Principle

### 3.1. Parameter Selection of Short Time Fourier Transform

Signals can be transformed into the frequency domain, sparse domain, or other combination domains for processing and analysis. Indistinct features in the time domain can be manifested through such transformation. The traditional Fourier transform is a global transformation based on the combination of different frequency components, which cannot express the time–frequency localization. In order to describe the time–frequency properties of signals, short-time Fourier transform (STFT) is proposed.

STFT is a joint time–frequency analysis method based on non-stationary signals. Its basic idea is to truncate the signal by a window function with a fixed length, and the Fourier transform is performed on each segment of the truncated signal to obtain the local frequency spectrum of each segment. Its model can be presented as [36]:

(5) $$S(\tau ,f)=\int x(t)w(t-\tau )e^{-2j\pi f\cdot t}dt$$

where *t* is the time, *f* is the frequency, x(t) is the time-domain signal, τ denotes a shift in time, and w(t−τ) is the window function, and *j* is an imaginary unit. By shifting ***τ*** continuously, Fourier Transforms at different times can be obtained. The set of these Fourier Transforms is *S*(*t*, *f*).

As an important processing tool in time–frequency analysis, the short-time Fourier transform has the advantages of simple principle and excellent localization. The weak local feature information can be captured by the two-dimensional representation of vibration signals in the time–frequency domain, and the high-dimensional spatial matrix is easier to leverage the ability of non-negative matrix decomposition algorithms, making compound faults diagnosis easier to implement.

Two main parameters (types and lengths of the window function) affect the effectiveness of the short-time Fourier transform. Window function is a method of truncating signals, which can reduce the effect of spectral leakage. The length of the window function affects the time–frequency resolution. The longer the window length, the higher the frequency resolution, but the time resolution is lower. Therefore, the type of window function and the length of the window need to be determined based on the specific signal type and processing environment.

**Figure 2:**
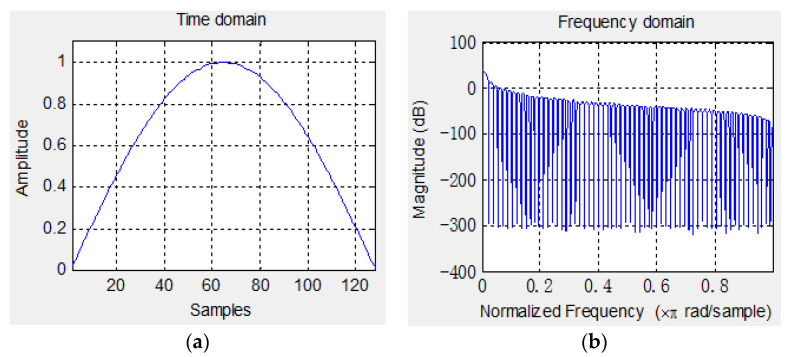
Example of Sine-bell window: (**a**) time-domain waveform; (**b**) the spectrum.

In order to reduce the effects of windowing and improve diagnostic accuracy, it is necessary to choose an appropriate window function. As we know, the wider the main lobe of the window function, the smoother the spectral peak of the signal is, and the more obvious the suppression effect of the fence effect is, but it will lead to a decrease in spectral resolution. From the perspective of spectrum analysis, it is required that the main lobe of the window function spectrum should be as narrow as possible to improve the resolution of the spectrum. At the same time, the side lobes of the window function spectrum should be as small as possible and decay rapidly with frequency, which can reduce leakage distortion. Therefore, comparing the performance of several common window functions for the coupling characteristics of compound fault signals in rotating machinery, the Sine-bell window is selected as the processing method in this paper. The sine-bell window performs well on side lobe suppression and can concentrate spectral energy in the main lobe. If the overlapping length is specified during its sliding process, the overlapping window segment can further compensate for signal attenuation at the window edge. The waveform and frequency response of the Sine-bell window are shown in Figure 2. The window length is 128 samples, and the overlap is half of the window length.

### 3.2. Multi-Constraint Non-Negative Matrix Factorization

The selection of the cost function for the non-negative matrix factorization algorithm is determined by the type of data and the application environment. Although NMF has been proven to be a useful tool in source separation, one drawback is that the separation performance tends to be poor in the case of noise. Moreover, NMF incurs a risk of degrading the separation performance in compound fault signals due to the lack of prior knowledge. Meanwhile, in the process of feature extraction for multi-source fault signals, the worse the correlation between source signals, the more obvious the locality displayed, and the better the effect on dimensionality reduction. On the contrary, there will be redundant components during the decomposition, which fails to describe the fault characteristics. Therefore, the dual constraints with *β*-divergence and determinant are selected as the cost function for the non-negative matrix factorization algorithm based on the characteristics of the fault signal. The *β*-divergence constraint can reduce limitations on data structures, and the determinant constraint can ensure the uniqueness of the base matrix ***W*** during the decomposition. The dual constraints can enhance local features effectively, which are more conducive to subsequent signal reconstruction. The model of *β*-divergence [37] can be presented as:

(6) $$d_{\beta }(y,x)=\{\begin{array}{c} \frac{y^{\beta }}{\beta (\beta -1)}+\frac{x^{\beta }}{\beta }-\frac{yx^{\beta -1}}{\left(\beta -1\right)}\;\;\;\;\;\;\beta \in R\backslash \{0,1\}\\ \;\;\;\;y\ln\frac{y}{x}-y+x\;\;\;\;\;\;\;\;\;\;\;\;\;\;\;\beta =1\\ \;\;\frac{y}{x}-\ln\frac{y}{x}-1\;\;\;\;\;\;\;\;\;\;\;\;\;\;\;\;\;\beta =0 \end{array}$$

From the above Equation (6), it is easy to prove the continuity about *β*-divergence when *β* = 0 and *β* = 1, and for any *β*, the following Equation (7) holds:

(7) $$d_{\beta }(\lambda y,\lambda x)=\lambda^{\beta }d(y,x)$$

When *β* = 0, it can be seen that Equation (7) has the property of scale invariance, which is independent of λ. The property of scale invariance indicates that energy components in the amplitude spectrum ***V*** have equal weight values during the decomposition. When *β* = 1, however, it overly relies on the higher energy components in the amplitude spectrum ***V***, which is not conducive to the separation of coupled signals. Therefore, *β* = 0 is chosen in this paper.

In order to ensure the uniqueness of the base matrix ***W*** and achieve better reconstruction results during the decomposition, the determinant constraint is introduced in the objective function of the NMF algorithm. The space formed by *n* m-dimensional column vectors $W_{1},W_{2},\ldots W_{n}$ is defined as ***P*(*W*)**, and the volume of ***P*(*W*)** can be represented as the following Equation (8):

(8) $$vol(P(W))=\{\begin{array}{c} \sqrt{\det(WW^{T})}\;\;\;\;(m<n)\\ \;\left|\det(W)\right|\;\;\;\;\;\;(m=n)\\ \sqrt{\det(W^{T}W)}\;\;\;(m>n) \end{array}$$

When vol(P(W)) is at its minimum value, the corresponding vector $W_{1},W_{2},\ldots W_{n}$ obtained can be determined uniquely.

The *β*-divergence constraint and determinant constraint are used as new objective functions for the non-negative matrix factorization algorithm, which can be represented:

(9) $$F(W,H)=d_{\beta =0}(V,WH)+\alpha •vol(P(W))$$

where α is the equilibrium parameter and is taken as 1 (α = 1) generally, which is used to balance the proportion of matrix ***W*** and the reconstruction error.

According to the gradient descent method, we derive the iterative update rule for the objective function as follows:

(10) $$W\leftarrow W\frac{[V•{\left(WH\right)}^{-2}]•H^{T}}{{\left(WH\right)}^{-1}•H^{T}}\;\;\;\;\;\;\;\;\;\;H\leftarrow H\frac{W^{T}[V{\left(WH\right)}^{-2}]}{W^{T}{\left(WH\right)}^{-1}}$$

When the objective function converges, the optimization with dual constraints can be achieved. The specific steps of Algorithm 1 are as follows:
**Algorithm 1** Multi-constraint Non-Negative Matrix Factorization*Step 1*. Initialize non-negative matrices ***W*** and ***H*** randomly*Step 2*. Calculate the initial value of the objective function according to Equation (9)*Step 3*. Solve and update the matrices ***W*** and ***H*** alternately and iteratively based on Equation (10)*Step 4*. If the objective function (Equation (9)) converges, the iteration process is stopped, and the matrices ***W*** and ***H*** are output; otherwise, steps (2) and (3) are performed once again

The advantage of the multi-constraint NMF algorithm is that the constraints of *β*-divergence and determinant are introduced in the objective function, which can be close to the source signal, and the redundant component is reduced during the decomposition.

### 3.3. Construction of Parameter WK

The kurtosis index is a numerical statistic that reflects the distribution characteristics of random variables. It is the normalized 4th-order center moment, which is a dimensionless parameter and is particularly sensitive to impact signals. The correlation coefficient can be characterized by the degree of similarity between two signals. Considering the advantages and disadvantages of two indicators, we constructed a comprehensive parameter called Weighted Kurtosis (WK) in this paper, which is defined as follows:

(11) WK=C•K

(12) $$C=\frac{E\left[\left(x-\bar{x})(y-\bar{y}\right)\right]}{E\left[{\left(x-\bar{x}\right)}^{2}\right]E\left[{\left(y-\bar{y}\right)}^{2}\right]}$$

(13) $$K=\frac{\sum_{i=1}^{n}{\left(x(i)-\bar{x}\right)}^{4}}{\sum_{i=1}^{n}{\left(x(i)-\bar{x}\right)}^{2}}$$

where *C* is the correlation coefficient between the signals *x* and *y*, and *E* represents the mathematical expectation, *K* is the Kurtosis value of the signal. According to the Schwartz inequality |C|≤1 can be inferred. Thus, the parameter *WK* can be seen as the weight of the Kurtosis value, called Weighted Kurtosis. We know that the early failures of rolling bearings are mostly characterized by impact, and kurtosis is used to detect the impact components in the reconstructed signal, while the correlation coefficient can be reflected in the correlation between the reconstructed signal and the original signal. Meanwhile, according to Equation (11), it can be seen when the signal is processed by the multi-constraint NMF algorithm; the larger the parameter WK in the reconstructed signal, the richer the feature information contained, which can represent the fault characteristic signal. Therefore, the parameter WK is constructed as a criterion for filtering the reconstructed signal in this paper.

## 4. Signal Separation Method Based on Multi-Constraint NMF

A separation method of multisource signals with multi-constraint non-negative matrix factorization is proposed for bearings in rotating machinery. The specific diagnosis steps of Algorithm 2 are summarized as follows:
**Algorithm 2:** Signal Separation Method Based on Multi-constraint NMF*Step 1.* The algorithm of the short-time Fourier transform (STFT) is performed to obtain a feature matrix with local information.*Step 2*. Take the square value of the feature matrix, and the multi-constraint NMF algorithm is used to reduce the dimension, and obtain the base matrix ***W*** and the coefficient matrix ***H***.*Step 3*. The matrix ***W*** and ***H*** are recombined in subspace, and the recombined signals with feature components in the time domain are obtained by the inverse short-time Fourier transform (ISTFT).*Step 4*. Calculate the WK values of the recombined signals *Step 5*. The separation signals with high WK values are selected for envelope spectrum analysis to extract the fault features of bearings.

**Figure 3:**
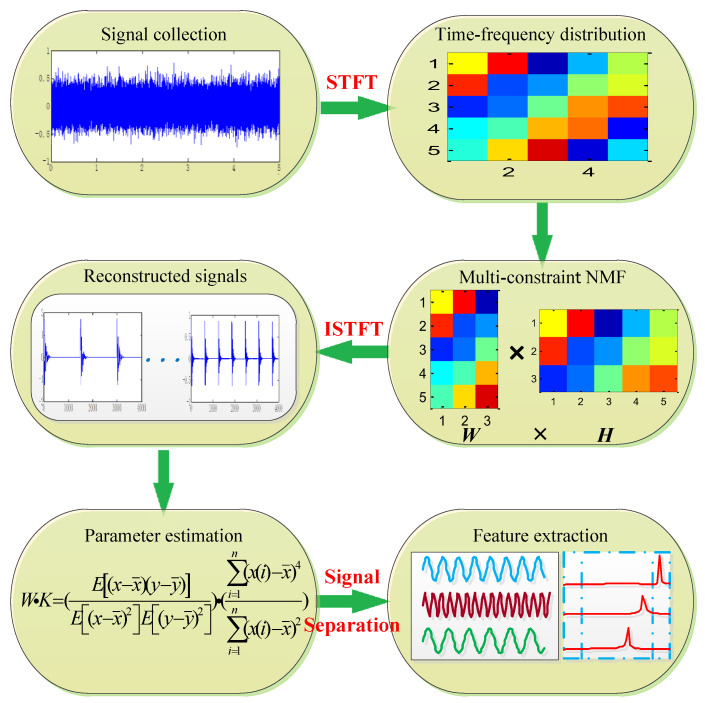
The flowchart of the proposed method.

The flowchart is presented in Figure 3.

## 5. Verification with Simulation and Experiment

### 5.1. Algorithm Simulation and Performance Analysis

**Figure 4:**
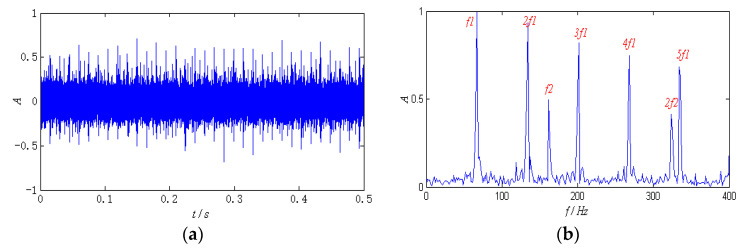
The simulated signal: (**a**) time-domain waveform; (**b**) the envelope spectrum.

In this section, the performance of the proposed multi-constraint algorithm is simulated and analyzed. The following model is applied to simulate compound faults in rolling bearing:

(14) $$s(t)=e^{-2\pi \zeta f_{n}(t-T)}\sin(2\pi f_{n}\sqrt{1-\zeta^{2}}(t-T))$$

(15) $$X(t)=As(t)=A{\left[s_{1}(t),s_{2}(t)\right]}^{T}+G(t)$$

where ***ζ*** is the damping coefficient, *s*_1_(*t*) and *s*_2_(*t*) are expressed as the following two feature parameters: The natural frequencies (*f_n_*) are 2500 Hz and 4500 Hz, respectively, and the characteristic frequencies (1/*T*) are 67 Hz and 162 Hz, the sampling frequency is 100 kHz, and the sampling data is taken as 0.5 s time segments. The mixed matrix ***A***(2 × 1) is generated randomly. The mixed source signal ***X***(***t***) is obtained by Equation (15), and ***G***(***t***) is Gaussian white noise (SNR = 5 dB) generated randomly. Figure 4 shows the mixed source signal and its normalized envelope spectrum.

**Figure 5:**
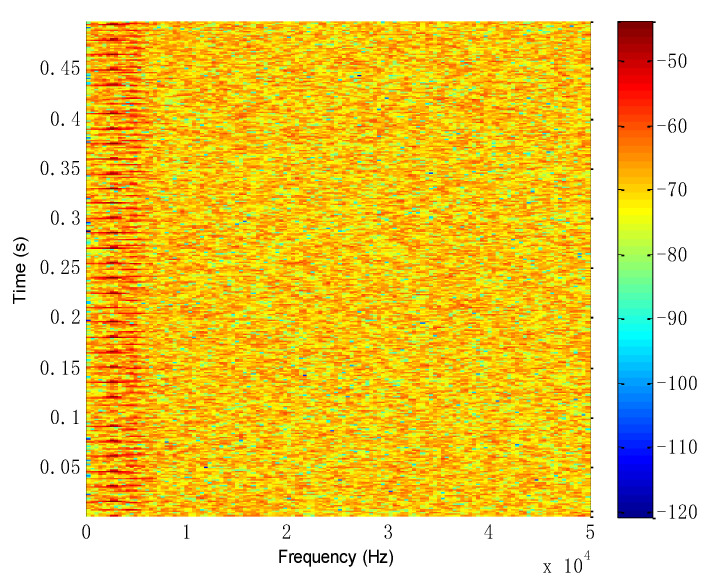
Time–frequency distribution of the simulated signal.

For the mixed source signals, the proposed method is performed for analysis. Firstly, the characteristic matrix ***M*** is obtained by the short-time Fourier transform, and the time–frequency distribution is shown in Figure 5. Secondly, the square value of the matrix ***M*** is obtained as the processing matrix of the multi-constraint NMF algorithm. Thirdly, the square-value matrix is decomposed by the multi-constraint NMF algorithm, and the base matrix ***W*** and the coefficient matrix ***H*** are obtained in dimensionality reduction. Finally, the obtained matrices are reconstructed by the inverse short-time Fourier transform in the subspace, presenting separated signals. Meanwhile, the WK values of the separated signals are shown in Table 1.

**Figure 6:**
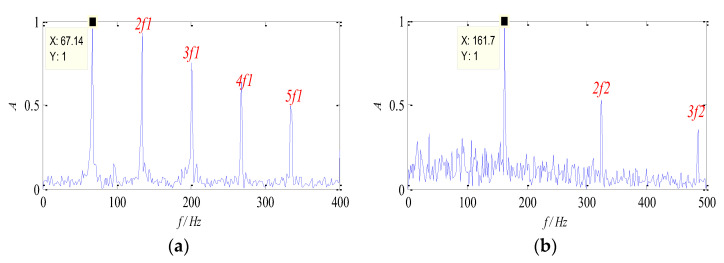
Envelope spectra of separated signal: (**a**) the signal ***s*_1_**; (**b**) the signal ***s*_2_**.

It can be seen from Table 1 that the WK values of Group 6 and Group 8 are relatively high, which indicates that the feature information in the two groups of signals is rich and describes the source signal better. The normalized envelope spectra of separated signals are shown in Figure 6. It is obvious that the two characteristic components (67 Hz and 162 Hz) can be separated by the proposed method, and their harmonic components are distinct, respectively. Therefore, it can be concluded that the proposed method can be used to separate the source signal from the mixed signals effectively, and the characteristic frequency can also be extracted in the envelope spectrum, which verifies the effectiveness of the proposed method.

### 5.2. Experimental Verification and Discussion

**Figure 7:**
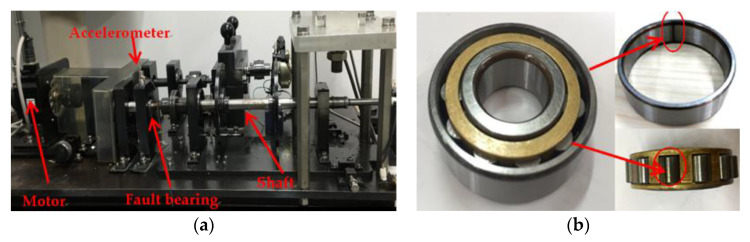
The experimental platform and fault bearing of simulation experiment: (**a**) experiment platform; (**b**) fault bearing.

In order to further validate the effectiveness of the proposed method, the measured compound fault signals of the roller bearing (N204) are used as the research object. The defects are machined artificially using the electrical discharge machining method on the outer ring and rolling elements of the bearing. The vibration signals in the vertical and horizontal directions are collected by the acceleration sensor (608A11). The platform of the simulation experiment and fault bearing are shown in Figure 7. The motor speed is set to 1300 rpm and 900 rpm, respectively, and the sampling frequency is 100 kHz (collect 100k sample points in 1 s). The sensor is set to collect data for 10 s. The fault passing frequency of rolling bearings can be calculated according to the structural parameters (Table 2). The theoretical characteristic frequency is shown in Table 3.

**Figure 8:**
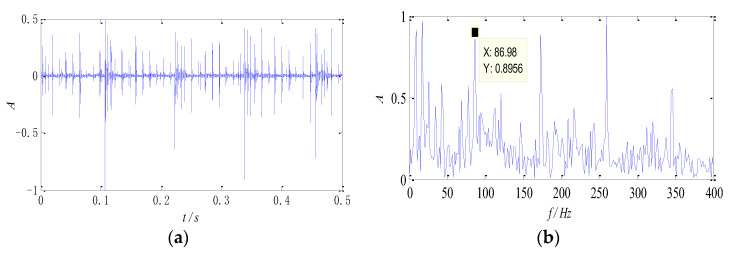
The signal of compound faults at 1300 rpm: (**a**) time-domain waveform; (**b**) the envelope spectrum.

The signals collected at 1300 rpm are used for analysis, and the data is taken as 0.5 s time segments randomly. The waveform and the normalized envelope spectrum of the signals are shown in Figure 8.

The impulse component can be seen clearly from the time-domain waveform, which indicates that the bearing has malfunctioned. The periodic property, however, is not obvious, and useful state information cannot be obtained. In the envelope spectrum, the defect feature of the outer race can be identified approximately, but the defect about the roller is submerged by the noise component and difficult to identify. In addition, peaks appear near 8 Hz and 16 Hz in the spectrum, which is close to the characteristic frequency of the cage and its second harmonic component, as well as the revolving frequency of the roller. The appearance of these two peaks may be caused by the impact of the rollers.

**Figure 9:**
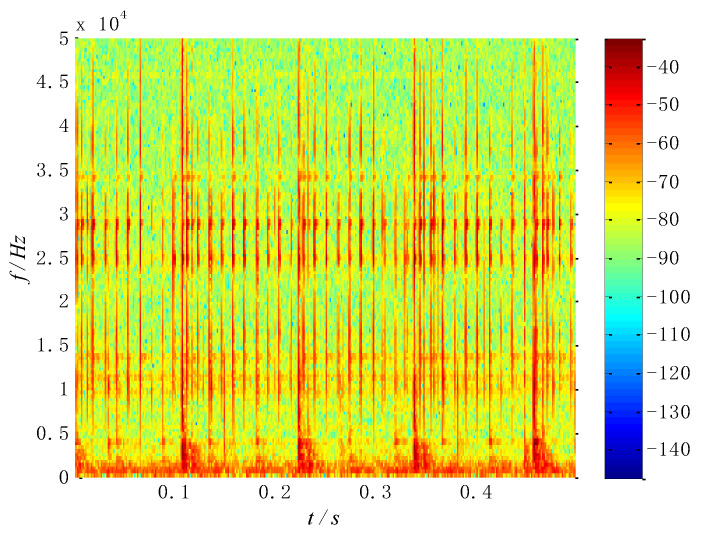
Time–frequency distribution of the collected signal at 1300 rpm.

According to the proposed method, the original signal is subjected to the short-time Fourier transform to obtain a feature matrix ***M***, and the time–frequency distribution is shown in Figure 9. The modulation and cluster of original signals can be seen clearly from the time–frequency distribution. The square value of the matrix ***M*** is obtained as the processing matrix of the multi-constraint NMF algorithm; after that, the square-value matrix is decomposed by the multi-constraint NMF algorithm to obtain the base matrix ***W*** and the coefficient matrix ***H***. Finally, the obtained matrices are reconstructed by the inverse short-time Fourier transform in the subspace, presenting separated signals. Meanwhile, the WK values of the separated signals are shown in Table 4.

**Figure 10:**
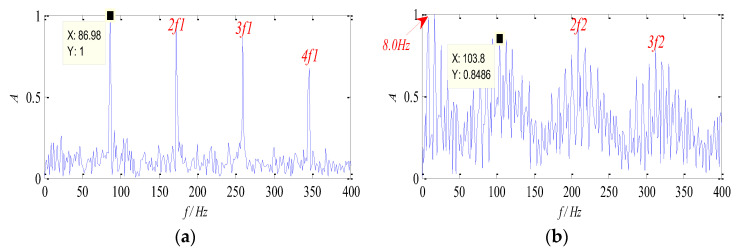
Envelope spectra of separated signals with the proposed method at 1300 rpm: (**a**) Envelope spectrum of outer-race fault; (**b**) envelope spectrum of roller fault.

It can be seen from Table 4 that the WK values of Group 2 and Group 7 are relatively high, which indicates that the feature information in the two groups of signals is rich and describes the source signal better. The normalized envelope spectra of separated signals are shown in Figure 10.

It is obvious that two leading constituents are obtained by the proposed approach, which accords with characteristic frequencies of the outer race and the roller. Meanwhile, their higher harmonic components are presented plainly. Furthermore, the feature frequency of the cage (8 Hz) and its high-frequency components appear in Figure 10b, and the sideband structure is protruded, which is in conformity with the roller failure. Therefore, the results indicate the effectiveness of the proposed approach, which can realize the separation of multi-source signals and their fault diagnosis of bearings.

**Figure 11:**
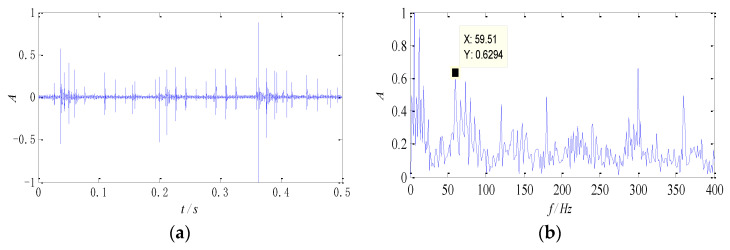
The signal of compound faults at 900 rpm: (**a**) time-domain waveform; (**b**) the envelope spectrum.

Similarly, the data is taken as 0.5 s time segments at 900 rpm randomly. The waveform and the normalized envelope spectrum of the signals are shown in Figure 11.

**Figure 12:**
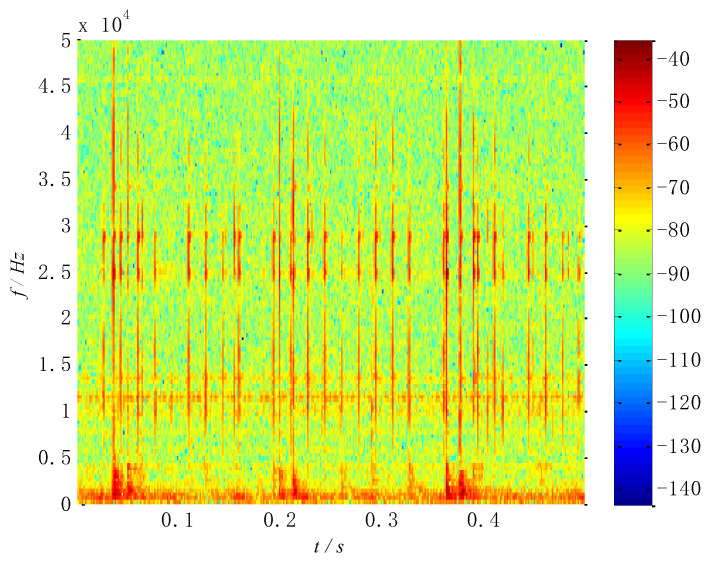
Time–frequency distribution of the collected signal at 900 rpm.

According to the proposed method, the time-frequency distribution is shown in Figure 12, and the WK values of the separated signals are shown in Table 5.

**Figure 13:**
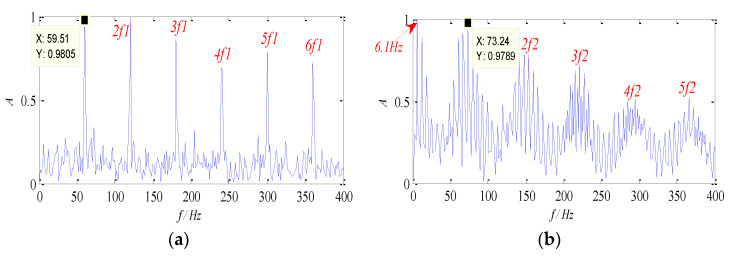
Envelope spectra of separated signals with the proposed method at 900 rpm: (**a**) Envelope spectrum of outer-race fault; (**b**) envelope spectrum of roller fault.

The separation signals with high WK values are selected for envelope spectrum analysis to extract the fault features of bearings and their normalized envelope spectra are shown in Figure 13.

Similarly, it is obvious that two leading constituents are obtained by the proposed approach, which accord with characteristic frequencies of the outer race and the roller. Meanwhile, their higher harmonic components are presented plainly. Furthermore, the feature frequency of the cage (6 Hz) and its high-frequency components appear in Figure 13b, and the sideband structure is protruded, which is in conformity with the roller failure. Therefore, the results support the effectiveness of the proposed approach in the field of compound fault diagnosis of bearings.

### 5.3. Comparison with Traditional Method

**Figure 14:**
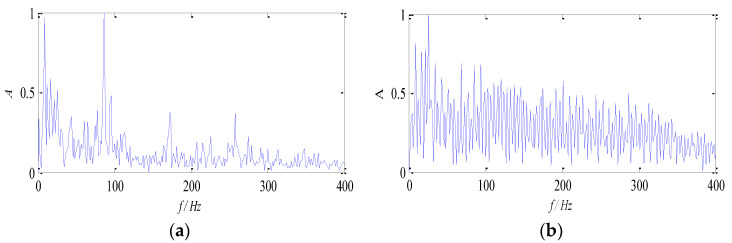
Envelope spectra of separated signals with the β-divergence method: (**a**) Envelope spectrum of *f*_1_; (**b**) envelope spectrums of *f*_2_.

**Figure 15:**
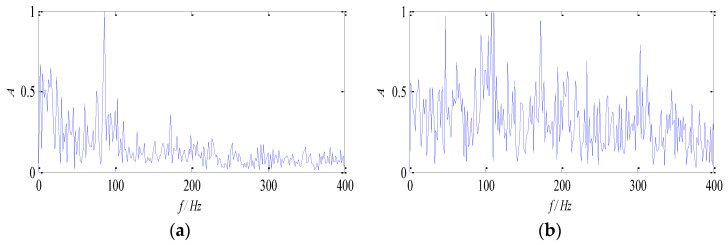
Envelope spectra of separated signals with the KL-divergence method: (**a**) Envelope spectrum of *f*_1_; (**b**) envelope spectrums of *f*_2_.

To demonstrate the advantages of the proposed method for multi-source signal separation, the traditional non-negative matrix factorization algorithm with β-divergence and KL-divergence are compared individually. The data at 1300 rpm is selected to illustrate it. The normalized envelope spectra of the separated signal are shown in Figure 14 and Figure 15.

It can be seen from Figure 14 and Figure 15 that the multi-source signals are not separated effectively with the traditional non-negative matrix factorization algorithm based on β-divergence and KL-divergence. The fault feature of the outer race is almost extracted, and the fault feature of the rolling element is submerged in environmental noise, which fails to describe the fault source signal accurately. Comparing traditional algorithms with the proposed algorithm, it can be seen that since the multi-constraint NMF algorithm enhances the local features of fault components, thus the multi-source signal can be separated, and the fault feature can be extracted.

## 6. Conclusions

In this paper, a novel blind source separation method under a single channel based on the multi-constraint NMF is proposed. The main research content and corresponding conclusions are as follows: (1) The performance of several common window functions are compared for compound fault signals, the Sine-bell window is selected as the processing method, and its parameter length is selected iteratively. (2) The constraints with *β*-divergence and determinant are introduced into the objective function of the traditional NMF algorithm, which can enhance local feature information and reduce redundant components during the decomposition. The iterative update rules for the multi-constraint NMF algorithm have been derived, and the convergence and practicality of the algorithm have been demonstrated in experiments. (3) The parameter Weighted Kurtosis (WK) is constructed as a criterion for filtering the reconstructed signals, and it has been proven to separate redundant signals effectively. (4) The simulated and experimental results indicate the effectiveness of the proposed approach, which realizes the separation of multi-source signals and extracts fault features. Meanwhile, compared with the NMF algorithm of the traditional objective function, the proposed method is more applicable for compound fault diagnosis.

It is worth considering that some deficiencies still exist, such as the initialization random of the algorithm in this paper. Therefore, future work will concentrate on the optimization initialization of the non-negative matrix factorization algorithm.

## Figures and Tables

**Table 1:** WK of the simulated signal.

	1	2	3	4	5	6	7	8	9	10
WK	6.52	3.78	2.74	9.08	2.96	31.74	3.96	22.28	2.89	6.54

**Table 2:** Structure parameters of N204 bearing.

Inner Diameter	External Diameter	Roller Diameter	Width	Number of Rollers
20 mm	47 mm	6.5 mm	14 mm	10

**Table 3:** Fault characteristic frequencies.

Fault Types	Outer Race	Roller	Cage
Characteristic frequencies at 1300 rpm	86 Hz	101 Hz	8 Hz
Characteristic frequencies at 900 rpm	60 Hz	74 Hz	6 Hz

**Table 4:** WK of the reconstructed signal at 1300 rpm.

	1	2	3	4	5	6	7	8	9	10
WK	0.51	88.72	0.48	0.47	0.28	0.27	211.52	0.83	0.63	2.79

**Table 5:** WK of the reconstructed signal at 900 rpm.

	1	2	3	4	5	6	7	8	9	10
WK	0.92	25.63	440.55	68.62	0.68	8.57	223.02	10.19	59.82	1.53

## Data Availability

The dataset generated and analyzed in the current study is available from the corresponding author upon reasonable request.
